# Extended Interportal Capsulotomy for Hip Arthroscopy, a Single-Center Clinical Experience

**DOI:** 10.3390/medicina60050738

**Published:** 2024-04-29

**Authors:** Ahmet Fırat, Enejd Veizi, Christos Koutserimpas, Hilmi Alkan, Ali Şahin, Şahan Güven, Yasin Erdoğan

**Affiliations:** 1 Department of Orthopedics and Traumatology, Ankara City Hospital, Ankara 06000, Turkey; ahmetfirat24@yahoo.com (A.F.); hilmi_alkan@hotmail.com (H.A.); dralisahin@yahoo.com.tr (A.Ş.); dr.sahanguven@gmail.com (Ş.G.); yasin-erdgn@hotmail.com (Y.E.); 2 Department of Anatomy, School of Medicine, Faculty of Health Sciences, National and Kapodistrian University of Athens, 75 Mikras Asias Str., Goudi, 11527 Athens, Greece

**Keywords:** hip surgery, arthroscopy, sports medicine, minimally invasive hip, hip injury, labrum

## Abstract

*Background and Objectives*: The number of hip arthroscopy procedures is on the rise worldwide, and awareness regarding proper management of the hip capsule has increased. No capsulotomy shape is agreed upon as a standard approach, with literature supporting both isolated interportal and T-shaped capsulotomies. The aim of this retrospective cohort study is to report the clinical results of a standardized extended interportal capsulotomy (EIPC) during hip arthroscopy. *Materials and Methods*: Patients operated on between 2017 and 2020 with a hip arthroscopy were eligible. The inclusion criteria were ages 18–60 years, failed non-operative treatment, and at least a 2-year follow-up. Exclusion criteria were bilateral femoroacetabular impingement syndrome (FAS) cases or labral lesions, ipsilateral knee injury, history of ipsilateral hip surgery, and significant spine lesions. Data regarding demographic characteristics such as age, gender, operation date, BMI, but also Beighton score, presence of postoperative pudendal nerve damage, and revision for any reason were gathered from patients’ records. All patients were evaluated preoperatively with a visual analog scale (VAS), the Hip Disability and Osteoarthritis Outcome Score (HOOS), and the modified Harris Hip Score (mHHS). *Results*: Of the 97 patients operated on with a hip arthroscopy between the defined dates, only 90 patients were included. The mean age was 37.9 ± 9.8, and 58.9% of patients were male. The most frequent surgical indication was an isolated FAS lesion (73.3%), followed by FAS associated with a labral tear (12.2%), an isolated labrum tear (10.0%), synovitis (3.3%), and a loose body (1.1%). The mean follow-up for the study cohort was 39.3 months. The majority of the patients had uneventful surgeries (76.7%), while there were three cases of sciatic nerve neuropraxia and 12 cases of pudendal nerve neuropraxia. Two patients underwent revision surgery during the study period. Comparison between preoperative and postoperative clinical scores showed a significant improvement with a final mHHS mean value of 67.7 ± 18.2, an HOOS value of 74.1 ± 13.2, and a low VAS score of 1.3 ± 1.2. *Conclusions*: A hip arthroscopy procedure with a standardized and unrepaired, extended interportal capsulotomy is a safe procedure with satisfactory mid-term results and high overall patient satisfaction. At a minimum of 2 years and a mean of 39.2 months, patients showed improved clinical scores and a low revision rate.

## 1. Introduction

The number of hip arthroscopy procedures has been increasing each year as more centers and physicians become aware of its advantages and become accustomed to the techniques [1,2]. A series of pathologies, such as labral tears, femoroacetabular impingement syndrome (FAS), arthritis, synovitis, loose bodies, septic arthritis, etc., previously requiring open surgery, are now mainly treated in a less invasive manner with good clinical results and relatively low complication rates [3,4].

Awareness regarding proper management of the hip capsule has increased lately, with the focus shifting from no repair at all to restoration of the native anatomy [5,6,7,8]. Numerous studies have reported the clinical benefits of repairing the capsule at the end of a hip arthroscopy while showing that an unrepaired capsule damages hip biomechanics [6]. The clinical impact of this is still uncertain, with some studies claiming that capsulotomy shape might have a final say [1,9,10].

To this date, no capsulotomy shape has been agreed upon as a standard approach for hip arthroscopy with literature supporting both isolated interportal capsulotomies and T-shaped capsulotomies [1,9,10,11]. Cadaveric studies have shown that a T-shaped capsulotomy offers greater visualization of the hip joint and head–neck junction, but this comes at the expense of having to perform a capsular repair [9]. An interportal capsulotomy, with a length of approximately 2–4 cm, is relatively easier to perform and repair but is reported to provide less visualization, potentially making it harder for surgeons to effectively approach periarticular pathologies [1,9,11]. A capsulotomy longer than 4 cm has been considered an extended capsulotomy, and the procedure has been the topic of previous cadaveric and clinical studies [9,10,11]. The aim of this study is to report the clinical results of a standardized arthroscopic extended interportal capsulotomy (EIPC) during the treatment of hip pathologies. We hypothesized that an unrepaired EIPC would show improved clinical results with comparable complications and revision rates in light of the current medical literature.

## 2. Materials and Methods

### 2.1. Patient Selection

This retrospective study included patients operated on at our Level I medical center between April 2017 and May 2020 and treated with hip arthroscopy. Inclusion criteria were patients treated with a hip arthroscopy for any diagnosis, between the ages of 18–60 with failed non-operative management (consisting of physical therapy and/or intra-articular injections), and with at least a 2-year clinical follow-up. Exclusion criteria were patients with bilateral FAS or labral lesions, ipsilateral knee injury, history of ipsilateral hip surgery, patients with a history of septic arthritis or cartilage damage, and with significant spine lesions (symptomatic radiculopathies, history of spine surgery, etc.) or other major medical comorbidities, refusal to participate, incomplete clinical data, and loss of follow-up. Surgery was indicated for cam, pincer, or mixed FAS lesions, labral tear (with or without a cyst), synovitis, septic arthritis, and the presence of a loose body within the joint.

Data regarding demographic characteristics such as age, gender, operation date, BMI, the Beighton score, and the presence of postoperative pudendal nerve damage and revision for any reason were gathered from patients’ records. Pudendal nerve damage was defined as postoperative hematoma associated with numbness or paresthesia in the groin or scrotolabial region. It was routinely noted on the first week postoperatively, together with the time it took to resolve or not. The Local Ethics Committee approved the study design (Approval No. E2-22-2121), and all patients gave their written and oral consent to participate in the study.

### 2.2. Surgical Procedure

**Figure 1:**
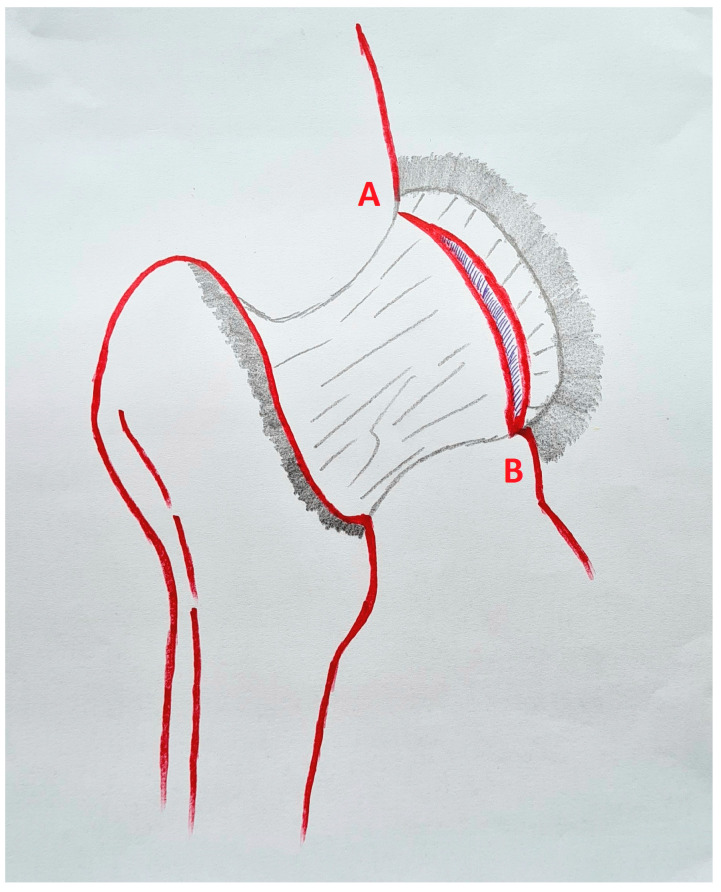
A schematic depiction of the extended interportal capsulotomy with an approximate length of 6–8 cm from point A to point B.

Surgeries were performed under epidural anesthesia and in a supine position. A traction table was routinely used, and all contact surfaces were well-padded to avoid potential injuries. After proper axial traction, an anterolateral (AL) portal was initially established. The anterior portal was then opened using a guidewire. Care was taken not to damage the labrum with either portal placement. A transverse interportal capsulotomy was then performed (Figure 1).

**Figure 2:**
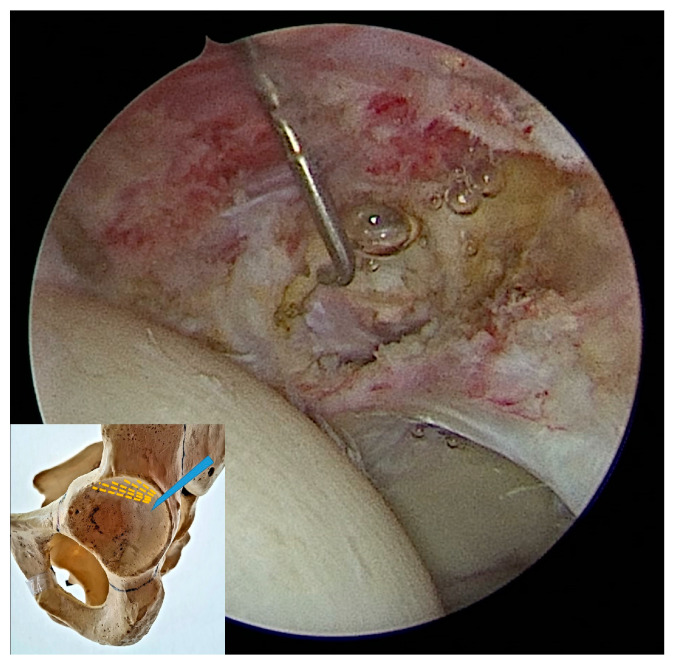
The proximal (anteromedial) end of the capsulotomy viewed from the anterolateral portal. The probe shows the iliopsoas tendon lying in the psoas valley.

**Figure 3:**
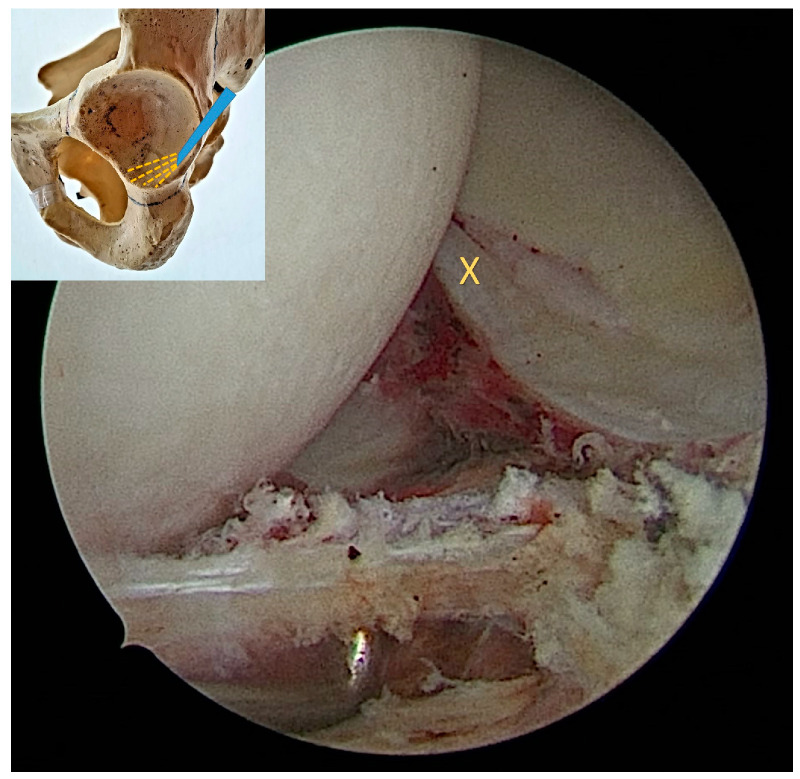
The distal (posteromedial) end of the capsulotomy viewed from the anterolateral portal. The beginning of the transverse acetabular ligament can be seen further distally (X).

Proximally, the capsulotomy was advanced up to the beginning of the psoas valley (Figure 2) and distally up to 1–2 cm from the beginning of the transverse ligament (Figure 3). This created a capsulotomy of 6–8 cm in length.

The capsule adjacent to the acetabulum was reflected, and the acetabular rim was exposed. The indexed intraarticular pathology was addressed, the acetabular rim was trimmed according to preoperative planning, and the labrum was reattached using suture anchors from a distal AL accessory (DALA) portal. Traction was then gently released until the hip could be brought to 30 degrees of flexion. The femoral neck was visualized distally, and capsular reflection was performed up until the intertrochanteric line. The femoral head-neck junction was visualized, and the indicated procedures between 12 o’clock and 6 o’clock, were then performed. A dynamic intraoperative examination was used after the completion of the procedures.

As a standard procedure, the capsulotomy was left unrepaired. The portals on the skin were sutured in a standard fashion, and the procedure was considered complete.

### 2.3. Rehabilitation Protocol

Patients were restricted to a partial weight-bearing protocol with crutches for the first 3 weeks, postoperative. A maximum of 90 degrees of hip flexion was allowed during this period, and hip extension and external rotation were discouraged. This was carried out to allow for the healing of the capsular repairs. We do not routinely use hip orthosis during our practice. Again, during the first 3 weeks after surgery, passive range of motion as well as passive hip circumduction exercises were allowed.

In the third week, muscle strengthening exercises as well as mild hip extension and external rotation were started. In the sixth week, closed kinetic chain exercises and stretching exercises were begun. Running was allowed after the twelfth week postoperatively, and a full return to sporting activities was slowly allowed between the sixth and eighth months postoperatively.

### 2.4. Clinical Follow-Up

All patients were evaluated preoperatively with a visual analog scale (VAS), the Hip Disability and Osteoarthritis Outcome Score (HOOS), and the modified Harris Hip Score (mHHS). The Beighton score was also preoperatively used to assess for generalized joint hypermobility [12]. Routine clinical visits were performed in the sixth postoperative month and at yearly intervals thereafter. Clinical score data was gathered on the final follow-up visit and was included in the final analysis. The gathering of clinical data was performed by an independent observer, not present during the surgical procedure.

### 2.5. Statistical Analysis

Statistical calculations were carried out using SPSS 25.0 (Chicago, IL, USA). Categorical variables are stated as numbers (n) and percentages (%), while continuous variables as mean ±, standard deviation (SD), and median (minimum-maximum). Comparison between preoperative and postoperative clinical scores was carried out using the Wilcoxon ranked test. G*Power software (v.3.1.9.7, Heinrich Heine Universitȁt Dȕsseldorf, Düsseldorf, Germany) was used to perform a post-hoc power analysis evaluating the strength of the study’s results. A *p*-value of <0.05 was considered statistically significant.

## 3. Results

**Figure 4:**
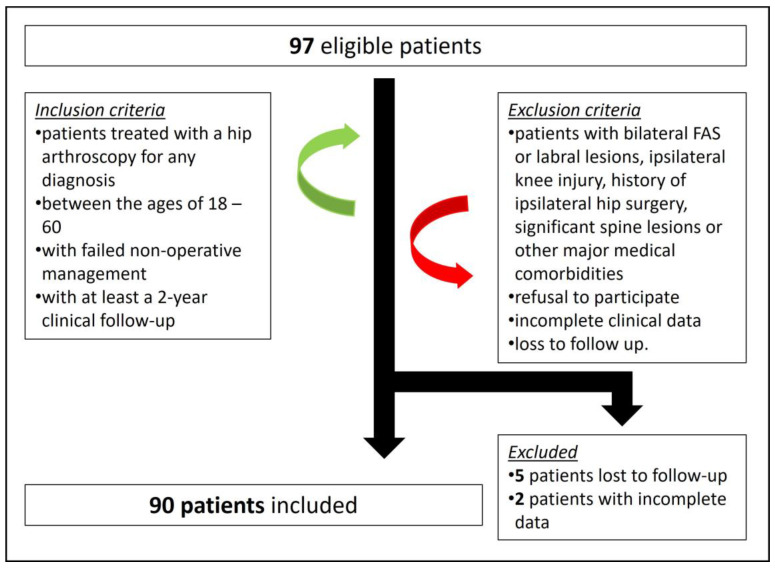
Inclusion and exclusion chart for the study’s cohort.

Of the 97 patients operated on with a hip arthroscopy between the defined dates, only 90 patients had complete follow-up data to be included in the study. Five patients were lost to follow-up, while 2 patients had no preoperative clinical score data (Figure 4).

The study cohort had a mean age of 37.9 ± 9.8 and 58.9% of patients were male. The most frequent surgical indication was an isolated FAS lesion (73.3%) followed by FAS associated with a labral tear (12.2%), isolated labrum tear (10.0%), synovitis (3.3%), and loose body (1.1%).

The mean follow-up for the study cohort was 39.3 months. The majority of the patients had uneventful surgeries (76.7%) while there were 3 cases of sciatic nerve neuropraxia and 12 cases of pudendal nerve neuropraxia. Thirteen of the patients regained their normal health within a week of the procedure while 2 patients had persistent complaints on their last follow-up visit (numbness around the groin area). Two patients underwent a revision surgery during the study period. One was due to a symptomatic heterotopic ossification of the superior capsule and the other was due to insufficient debridement of the Cam lesion. All other demographic data is presented in Table 1.

Comparison between preoperative and postoperative clinical scores showed a significant improvement with a final mHHS mean value of 67.7 ± 18.2, an HOOS value of 74.1 ± 13.2, and a low VAS score of 1.3 ± 1.2. All data regarding clinical scores is presented in Table 2. A post-hoc power analysis, evaluating the strength of these results showed a study power of 99–100%.

## 4. Discussion

Hip arthroscopy is a reliable procedure in the treatment of FAS and labral tears but is only possible through a capsulotomy, performed to gain access to the central and peripheral compartments of the hip joint [13]. Despite this, substantial controversy exists regarding the type of capsulotomy, and whether to repair the capsule at the end of the procedure [14]. This single-center, single-surgeon retrospective study reported the clinical results of ninety consecutive hip arthroscopies with a minimum follow-up time of 2 years and unrepaired capsules at the end of the procedure. The results showed improved clinical scores as a mean of 39.3 months with a low complication rate. A routine extended interportal capsulotomy without a capsular repair was used throughout the study and no revision surgery was needed for capsule-related issues.

The overall number of hip arthroscopies performed worldwide has surged in the last decade [4,15]. The surge is due not only to the increased familiarity with the technique and the equipment but also to the awareness that pathologies around the hip joint have gained [16,17]. A painful hip is most commonly a result of femoroacetabular impingement syndrome (FAS), labral tears, snapping hip syndrome, osteoarthritis, synovitis, a loose body, or septic arthritis [17,18]. Open procedures have been replaced by less invasive arthroscopic interventions and a recent systematic review by Kyin et al. [18] reported considerable improvement in patient-reported outcomes in the mid- to long term. The results of this study were compared favorably with the literature. Our cohort of 90 patients showed significant improvement at a mean follow-up of 39.3 months with an overall low revision rate.

Revision after hip arthroscopy is less frequent compared to the previous decade [18] and the evolving approach to capsular management has played an important role in it [17]. Exposure of the hip joint requires almost always a capsulotomy, which in most cases is longer than 2 cm and incises the iliofemoral ligament, if not cutting it completely [9,10,17]. There is an ongoing debate on whether this lays the grounds for instability in an otherwise inherently stable joint, and therefore should be kept at a minimum size, or that its clinical significance is poor given the ability to surround tissue to heal over time [1]. The most commonly used capsulotomies are the interportal capsulotomy (standard or extended) and the T-shaped capsulotomy [11,17].

An interportal capsulotomy has an average length of 2–4 cm, is relatively easier to perform and to repair, but is reported to provide less visualization, potentially making it harder for surgeons to effectively approach periarticular pathologies, especially in less experienced hands [9,10]. A capsulotomy longer than 4 cm (often 6–8 cm) has been considered an extended interportal capsulotomy. Cvetanovich et al. [9], in their anatomical study, found that a T capsulotomy resulted in similar visualization compared with a 6 and 8 cm extended interportal capsulotomy. Weber et al. [10] on the other hand reported that both extended interportal and T-capsulotomies resulted in equivalent hip distraction and they suggested that both be repaired for more anatomical healing. Clinically both capsulotomies provide sufficient visualization since the intraoperative joint is a dynamic structure and instrumentation can increase the reachable cross-section area without the need for a ‘T’ arm in the capsule. The low revision rate of this study reflects the fact that an extended capsulotomy is at least as good as a T-shaped one. The topic is still controversial in the literature. In their meta-analysis study, Lin et al. [19] reported comparable revision and complication rates between interportal and T-shaped capsulotomies and concluded that the currently published evidence was still not strong enough to confirm the superiority of repairing the capsule after hip arthroscopy. On the other hand, additional studies have reported a revision rate ranging from 1.2% to 13% after both capsulotomies [6].

Prolonged traction time during hip arthroscopy has been associated with a series of complications such as sciatic nerve neuropraxia [20], pudendal nerve damage [21], groin or labial hematomas [22], pressure necrosis [16], and even avascular necrosis [23]. Similarly, most of the complications of this cohort were traction-related and transient, with only 2 patients (2.2%) having persistent complaints. This is compatible with the overall reported incidence of neurological complications in the literature, ranging from 1.4% to 13% [24,25].

Management of the capsulotomy is another significant factor that has evolved over time [1,17]. Freeman et al. [17] among many others have shown that repairing the capsule after the procedure restores biomechanical properties of the joint to almost native levels [1,9]. Domb et al. [26] found no difference in clinical outcomes at a 2-year follow-up in patients receiving a capsular repair versus those without. They later reported that patients without a capsular repair had higher rates of conversions to a total hip and lower mHHS at a minimum of 5 years after surgery. Also, Bolia et al. [27] reported that patients without a capsular repair after a hip arthroscopy were 6.8 times more likely to convert to a hip replacement at a minimum follow-up of 6.4 years. Patients in our study did not undergo a capsular repair; nevertheless, overall clinical scores at a minimum of 2 years postoperatively show a significant improvement. The reason this cohort did not undergo capsular repair has to do with the senior surgeon’s preference at the time and the ambiguity surrounding the topic during the last decade. Recently and continuously, the importance of capsular repair has led to a change in our clinical approach with routine capsular repair now being performed after every case.

The positive results of this study could be attributed to the performance of a standardized procedure. Some surgical tips worth mentioning could be that the capsule can be opened anteromedially up to the iliopsoas. However, the iliopsoas valley should not be entered to prevent retroperitoneal fluid leakage. If tenotomy is necessary, it should be postponed until the end of the procedure. Also, the capsulotomy should not be extended more posteriorly. This situation can lead to major or minor complications, especially in patients with dysplasia. Additionally, the capsule remaining on the acetabular side can be thinned in order for the capsulolabral interval to be opened to reach the acetabulum. During thinning, total resection of the capsule should be avoided, as this may affect postoperative recovery or, conversely, releasing too much of the capsule may cause impingement of the capsule in advanced flexion degrees postoperatively. Finally, during the thinning of the femoral side of the capsule to reach the cam lesion, extra care should be taken not to damage the zona orbicularis so that postoperative hip joint stability can be retained. Additionally, since the capsule is left unrepaired, we suggest it be considered carefully in patients with borderline dysplasia since it might constitute a possible contraindication. The addition of a periacetabular osteotomy in these cases might help. Another factor that might have enhanced the chances of success is the postoperative rehabilitation protocol. Patients were allowed partial weight-bearing with crutches for the first postoperative 3 weeks and a maximum of 90 degrees of hip flexion was allowed during this period to allow for capsular healing.

Despite its positive results, this study should be appraised considering its limitations. This was a single-center, single-surgeon retrospective cohort study, and the results might not reflect the general practice of the field. Standardization of the procedure, surgical experience, and an accustomed surgical team might have led to better overall results compared to centers with a lower volume of patients requiring a hip arthroscopy. Another limitation of the study is the heterogeneity of the study group created by the inclusion of all patients operated on and the possibility of a type two error (selection bias) during inclusion. A cohort with similar and narrow surgical indications would have yielded more valuable information. An additional limitation of our study is the lack of a control group making it impossible to prove the real impact of a repaired extended interportal capsulotomy on patients undergoing a hip arthroscopy. Also, among the exclusion criteria, we have not recorded data regarding any internal diseases (such as diabetes) or other factors (such as smoking or alcohol abuse) that have been showed to affect the healing of tissues after arthroscopic procedures, including the formation of a scar after capsulotomy and even avascular necrosis [23,28]. Furthermore, clinical results were presented as preoperative and postoperative clinical scores, and traction time along with overall surgical time was not recorded. Additional information, such as tests for instability, data regarding range of motion, and overall traction time would have made the study more valuable. Also, although we did not have such complications, postoperative laxity, subluxation, dislocation, and revisions have been reported after unrepaired capsulotomy and surgeons should be aware of the risks involved, especially during the learning curve of the procedure. Finally, the stability of extended capsulotomy would have been further strengthened by postoperative images such as MRI or CT arthrograms, in addition to clinical outcomes, data which was not evaluated for this study. Nevertheless, the results of this study show that a hip arthroscopy procedure performed with a standardized and then repaired capsulotomy yields good clinical results and a low revision rate at a mid-term follow-up.

## 5. Conclusions

A hip arthroscopy procedure with a standardized and unrepaired, extended interportal capsulotomy is a safe procedure with satisfactory mid-term results and high overall patient satisfaction. At a minimum of 2 years and a mean of 39.2 months, patients showed improved clinical scores and a low revision rate.

## Figures and Tables

**Table 1:** Demographic characteristics of the study cohort.

	Patients (n = 90)
**Age**	
Mean ± SD Median (Min–Max)	37.9 ± 9.8 37 (17–60)
**Side**	
Right Left	58 (64.4%) 32 (35.6%)
**Gender**	
Male Female	53 (58.9%) 37 (41.1%)
**BMI**	
Mean ± SD Median (Min–Max)	25.2 ± 2.9 25 (17–32)
**Diagnosis**	
FAS	66 (73.3%)
FAS + Labrum tear	11 (12.2%)
Isolated labrum tear	9 (10.0%)
Synovitis	3 (3.3%)
Loose body	1 (1.1%)
**Beighton**	
0	71 (78.9%)
1	15 (16.7%)
2	3 (3.3%)
3	1 (1.1%)
**Follow-up (months)**	
Mean ± SD Median (Min–Max)	39.3 ± 14.0 36 (24–61)
**Complications**	
None	69 (76.7%)
Sciatic nerve neuropraxia	3 (3.3%)
Pudendal nerve neuropraxia	12 (13.3%)
Groin/Labial hematomas	2 (2.2%
Heterotopic ossification	2 (2.2%)
Iatrogenic chondral lesion	1 (1.1%)
Hardware breakage	1 (1.1%)
Infection	0 (0.0%)
**Revision**	
No Yes	88 (97.8%) 2 (2.2%)

**Table 2:** Comparison of preoperative and postoperative clinical scores.

	Preoperative	Postoperative	*p*-Value *
Modified Harris Hip Score (mHHS)	58.4 ± 9.4 57.0 (40–88)	67.7 ± 18.2 70.0 (29–98)	0.000
Hip Disability and Osteoarthritis Outcome Score (HOOS)	57.2 ± 13.8 61.0 (19–90)	74.1 ± 13.2 78.6 (42–96)	0.000
Visual Analog Scale (VAS)	4.6 ± 1.3 4 (3–9)	1.3 ± 1.2 1 (0–5)	0.000

* Wilcoxon Ranked test.

## Data Availability

Data is available upon reasonable request from the corresponding author.
